# CsoR Is Essential for Maintaining Copper Homeostasis in *Mycobacterium tuberculosis*

**DOI:** 10.1371/journal.pone.0151816

**Published:** 2016-03-21

**Authors:** Sarah A. Marcus, Sarah W. Sidiropoulos, Howard Steinberg, Adel M. Talaat

**Affiliations:** Department of Pathobiological Sciences, University of Wisconsin-Madison, Madison, Wisconsin, United States of America; Infectious Disease Research Institute, UNITED STATES

## Abstract

*Mycobacterium tuberculosis*, a pathogen infecting one third of the world population, faces numerous challenges within the host, including high levels of copper. We have previously shown that *M*. *tuberculosis* CsoR is a copper inducible transcriptional regulator. Here we examined the hypothesis that *csoR* is necessary for maintaining copper homeostasis and surviving under various stress conditions. With an unmarked *csoR* knockout strain, we were able to characterize the role of *csoR* in *M*. *tuberculosis* as it faced copper and host stress. Growth under high levels of copper demonstrated that *M*. *tuberculosis* survives copper stress significantly better in the absence of *csoR*. Yet under minimal levels of copper, differential expression analysis revealed that the loss of *csoR* results in a cell wide hypoxia-type stress response with the induction of the DosR regulon. Despite the stress placed on *M*. *tuberculosis* by the loss of *csoR*, survival of the knockout strain was increased compared to wild type during the early chronic stages of mouse infection, suggesting that *csoR* could play an active role in modulating *M*. *tuberculosis* fitness within the host. Overall, analysis of CsoR provided an increased understanding of the *M*. *tuberculosis* copper response with implications for other intracellular pathogens harboring CsoR.

## Introduction

*Mycobacterium tuberculosis* is one of the world’s most successful bacterial pathogens, infecting approximately one third of the human population. Despite widespread vaccination and the existence of antibiotic therapies, this causative agent of tuberculosis leads to over 1 million deaths each year [1]. Part of the success of this pathogen is attributed to its ability to quickly adapt and survive within harsh host microenvironments. The stress conditions *M*. *tuberculosis* must face within the host include reactive nitrogen and oxygen species [2], low pH [3], and hypoxia [4]. Recently, copper has been recognized as an additional weapon in the host macrophage’s arsenal as it localizes high levels of copper to the mycobacterial phagosome [5]. This finding led our group to investigate the *M*. *tuberculosis* response to copper stress revealing a set of 30 genes responsive to copper and the damage it can cause [6]. Among these genes were two that encode for copper-responsive transcriptional repressors–paralogs now identified as *ricR* and *csoR*–the latter of which we continue to characterize in this report.

The bactericidal capabilities of copper have been known for some time [7]. The mechanisms of copper damage include production of oxidative stress through Fenton reactions [8], displacing metal cofactors in proteins, and destabilization of Fe-S clusters [9]. Indeed, high physiological levels of copper have been demonstrated to be bactericidal for *M*. *tuberculosis* [5, 6]. Conversely, copper serves as a cofactor of metalloenzymes and is necessary in trace amounts for various cellular activities [10]. In *M*. *tuberculosis* such metalloenzymes include cytochrome *c* oxidase and superoxide dismutase [11], thus the role of copper in metabolism and the copper stress response must be carefully regulated. For example, under hypoxic conditions, which increase copper toxicity, *M*. *tuberculosis* makes use of copper-independent cytochrome *bd* oxidase [12]. This effectively reduces the requirement for cytochrome *c* oxidase, and therefore copper, under conditions where copper poses the greatest threat to the cell.

To better understand how the copper stress response is managed, we began to characterize one *M*. *tuberculosis* copper-induced regulator, CsoR. The crystal structure of CsoR revealed that the protein forms a homodimer with each monomer binding one molar equivalent of Cu(I) [13]. In its apo form, CsoR binds the promoter of its own copper-sensitive operon (*cso*), however upon binding copper, repression is released inducing expression of the *cso* in a graduated manner leading to the export of copper ions. Here we investigated the importance of the negative regulatory role of *csoR* in *M*. *tuberculosis* copper homeostasis. Our initial results, examining the Δ*csoR* strain under copper stress and during early chronic murine infection, suggested that under certain conditions *M*. *tuberculosis* may have an advantage upon suppressing the expression of *csoR*. Closer examination of the transcriptome of *M*. *tuberculosis* lacking *csoR*, however, revealed a hypoxic stress response during growth in copper-free media. This response may have better prepared the bacilli for survival during the early chronic stage of murine infection. We expect that *M*. *tuberculosis* must strike a balance when it comes to handling copper stress and that CsoR is integral, though perhaps not alone, in this role. Furthermore, in this role CsoR may make an important contribution during entry to the chronic phase of tuberculosis.

## Results

### Generation of *M*. *tuberculosis csoR* constructs

**Fig 1:**
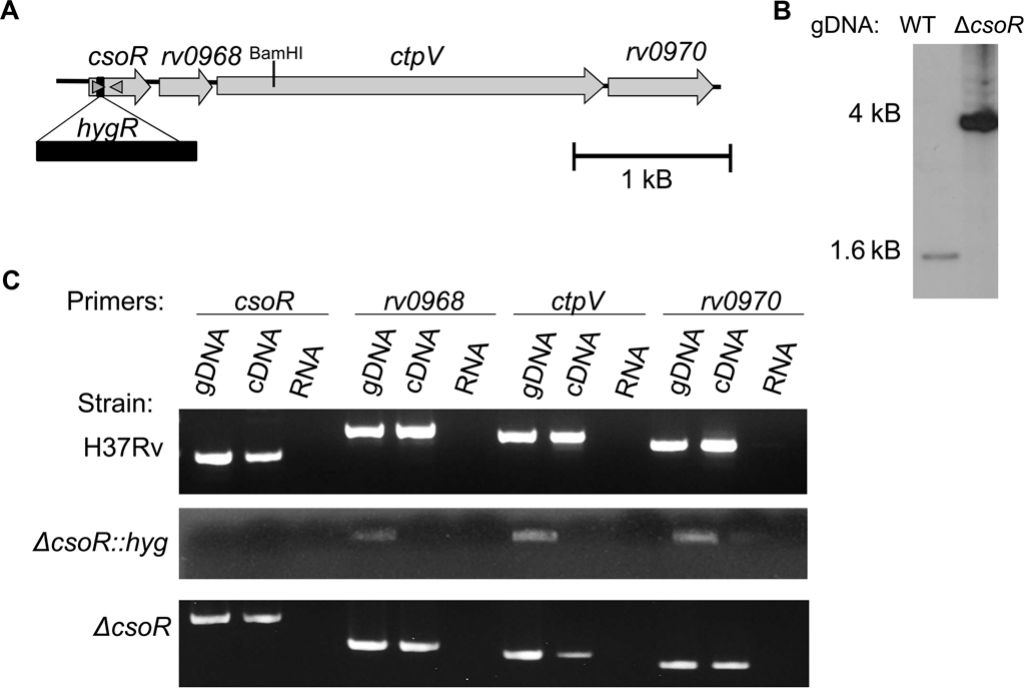
Construction of a nonpolar *M*. *tuberculosis* Δ*csoR* strain. **(A)** Diagram showing the copper-sensitive operon drawn to scale. To knock out *csoR*, the first gene of 4 in its operon, a 2.4kB *hyg*_*R*_ cassette (black bar) was inserted near the 5’ region of *csoR*. Grey arrowheads indicate primers flanking the genomic region amplified to be used as a probe for Southern blot. The BamHI cut site within the *cso* used for Southern blot is shown. **(B)** Southern blot targeting the *csoR* region after BamHI digestion of wild type (WT) or Δ*csoR*::*hyg*_*R*_ genomic DNA (gDNA) demonstrating insertion of the *hyg*_*R*_ cassette. **(C)** The polar nature of the different Δ*csoR* constructs was tested by RT-PCR of H37Rv, Δ*csoR*::*hyg*_*R*_, and Δ*csoR* after exposure to 500μM CuCl_2_, with gDNA positive and RNA negative controls from the same strains.

To create a knockout mutant of *csoR* in the virulent, wild type *M*. *tuberculosis* strain H37Rv, homologous recombination was used to replace 5 base pairs (bp) of the 360bp coding region of *csoR* with a hygromycin resistance cassette (*hyg*_*R*_), an ~2.4kB region, using the cosmid pYUB854 (Fig 1A) [14]. The mutant was confirmed by sequencing and with Southern blot, which showed a 2.4kB shift between bands from wild type or mutant strains (Fig 1B). However, reverse transcriptase PCR (RT-PCR) testing for expression of the downstream genes revealed loss of transcription of all three downstream genes in the *cso*: Rv0968, a conserved hypothetical protein; *ctpV*, coding for a copper-exporter [15]; and Rv0970, a conserved membrane protein of unknown function (Fig 1C). In order to create a nonpolar Δ*csoR* strain, an additional step was taken to remove the hygromycin cassette. The vector pYUB870 [14] was electroporated into Δ*csoR*::*hyg*_*R*_ removing *hyg*_*R*_ and leaving behind a 150 bp insertion sequence at the 14^th^ codon of *csoR*. The resulting unmarked mutation led to a frame shift which introduced 6 stop codons either at the insertion site or within the remainder of the *csoR* gene. RT-PCR results confirmed transcription of the remaining members of the *cso* in the unmarked mutant strain (Fig 1C). A complementation strain, Δ*csoR*::*csoR*, was constructed by incorporating *csoR* under its own promoter into the Δ*csoR* genome using the integrative vector pMV306.

To ensure that no other possible disruptions of the genome in or outside of the *cso* were contributing to the examined phenotypes, whole genome sequencing was carried out comparing *M*. *tuberculosis* Δ*csoR* with its wild type H37Rv parent strain. A total of 95 single nucleotide polymorphisms (SNPs) were found in *M*. *tuberculosis* Δ*csoR* when compared to the published H37Rv reference [16]. Of these, 38 SNPs corresponded to those identified as sequencing errors in the original reference [17] and 49 of the remaining SNPs were found in the sequenced parent strain. In all 8 unique SNPs were found in *M*. *tuberculosis* Δ*csoR* differentiating it from the parent strain. Two of these SNPs had been previously identified as they were annotated at the start of the insertion sequence in *csoR*. Five of the other six SNPs were found to be non-synonymous. One SNP each was found in PPE5 (Rv0304c) and PPE55 (Rv3347c). Three more, including the one synonymous SNP, were found in PE_PGRS9 (Rv0746). The last was found in a probable cyclase, Rv2435c. When *M*. *tuberculosis* Δ*csoR* was searched for larger genomic variations, only the 150bp scar at the *csoR* deletion site was identified. Given the distance of these variations from the *cso* and the function of the genes they target, it seems unlikely that the observed phenotypes could be due to anything other than the deletion of *csoR*.

### Survival of Δ*csoR* is enhanced under copper stress

**Fig 2:**
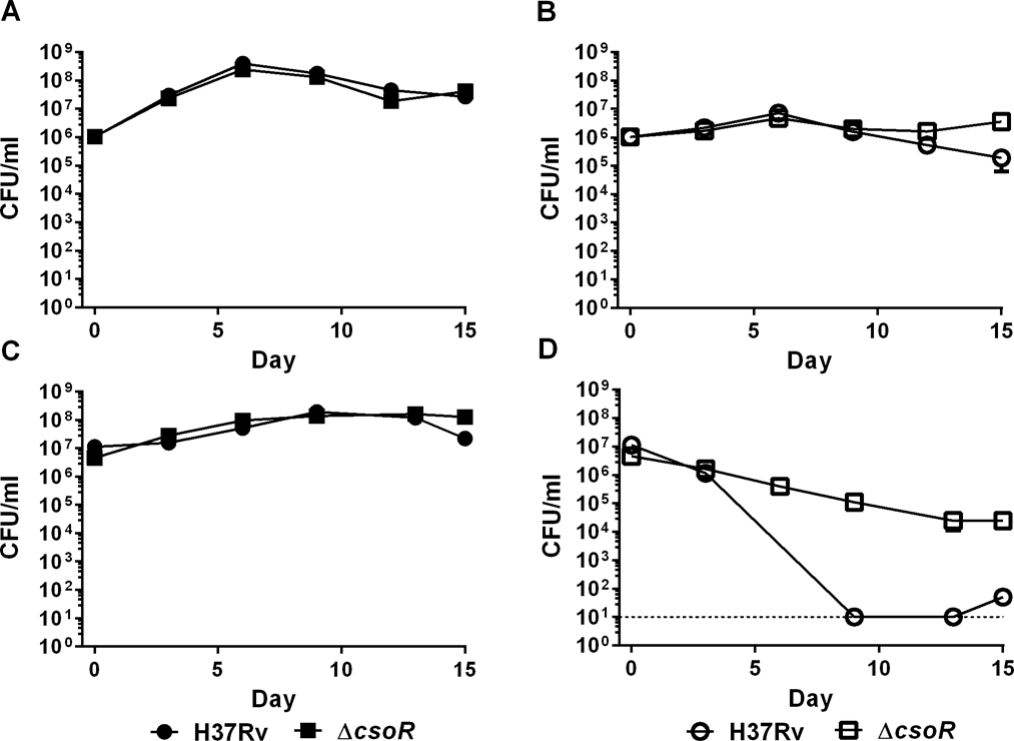
Growth kinetics of Δ*csoR* under copper stress. **(A)** Growth of *M*. *tuberculosis* H37Rv (circles) and Δ*csoR* (squares) over the course of 15 days in Sauton’s media left untreated (filled), or **(B)** treated with 500μM CuCl_2_ (open). **(C)** Growth of stationary phase *M*. *tuberculosis* H37Rv (circles) and Δ*csoR* (squares) inocula over the course of 15 days in Sauton’s media left untreated (filled), or **(D)** treated with 500μM CuCl_2_ (open). The dashed line indicates the limit of detection. Shown are one of two similar biological replicates with error bars representing standard deviation.

To test the growth kinetics of *M*. *tuberculosis* Δ*csoR* during copper stress, wild type and mutant strains were grown in copper-free Sauton’s liquid media with no added copper or with 50 or 500μM CuCl_2_. As expected, both strains showed similar levels of growth in untreated cultures suggesting no generalized growth defects (Fig 2A), and showed a decline in growth in the presence of 500μM CuCl_2_ (Fig 2B). While the gradual decline of the *ΔcsoR* strain in 500μM CuCl_2_ was similar to wild type at early time points, by day 15 the culture of the knockout strain maintained its total CFU/ml while the wild type culture continued to decline (Fig 2B). The difference at this time point was significant (*P* = 0.002) and observed across two separate experiments. Growth of the two strains in 50μM CuCl_2_ did not vary from that of untreated cultures (S1 Fig).

To investigate the possibility that *csoR* and its regulon may play a greater role during later stages of growth as the culture transfers into stationary phase, the growth curves were repeated inoculating from cultures that had reached stationary phase, rather than from actively growing cultures as was done above. Interestingly, the difference in survival between *M*. *tuberculosis* wild type and *ΔcsoR*, while not affected in untreated cultures (Fig 2C), differed dramatically in the presence of 500μM CuCl_2_ (Fig 2D). This difference was noted after day 4 when wild type survival fell two logs lower than the mutant. By day 8 it remained near the limit of detection, 10 CFU/ml, while *ΔcsoR* survival declined much more gradually, remaining above 10^4^ CFU/ml through day 15, indicating the ability of *ΔcsoR* to adapt to increased Cu levels.

After testing survival under two concentrations of copper, we sought to investigate the impact of copper on the growth of *M*. *tuberculosis* Δ*csoR* at a higher resolution. This was done by employing a modified microplate Alamar blue assay (MABA) [18, 19]. Growth inhibition of *M*. *tuberculosis* wild type H37Rv, *ΔcsoR*, and Δ*csoR*::*csoR* by two-fold dilutions of CuCl_2_ ranging from 16μM to 4000μM was tested (S2 Fig). Microplates were inoculated with cultures growing at mid-log phase and read after approximately 10 days of growth. Susceptibility of both wild type and complement strains was equivalent, both showing a minimum inhibitory concentration (MIC) of 250μM CuCl_2_. The mutant, on the other hand, showed an MIC of 500μM CuCl_2_. These data confirmed the idea that loss of *csoR* improves survival of *M*. *tuberculosis* under copper stress.

### Absence of *csoR* improves survival of *M*. *tuberculosis* during the early stages of chronic infection

**Fig 3:**
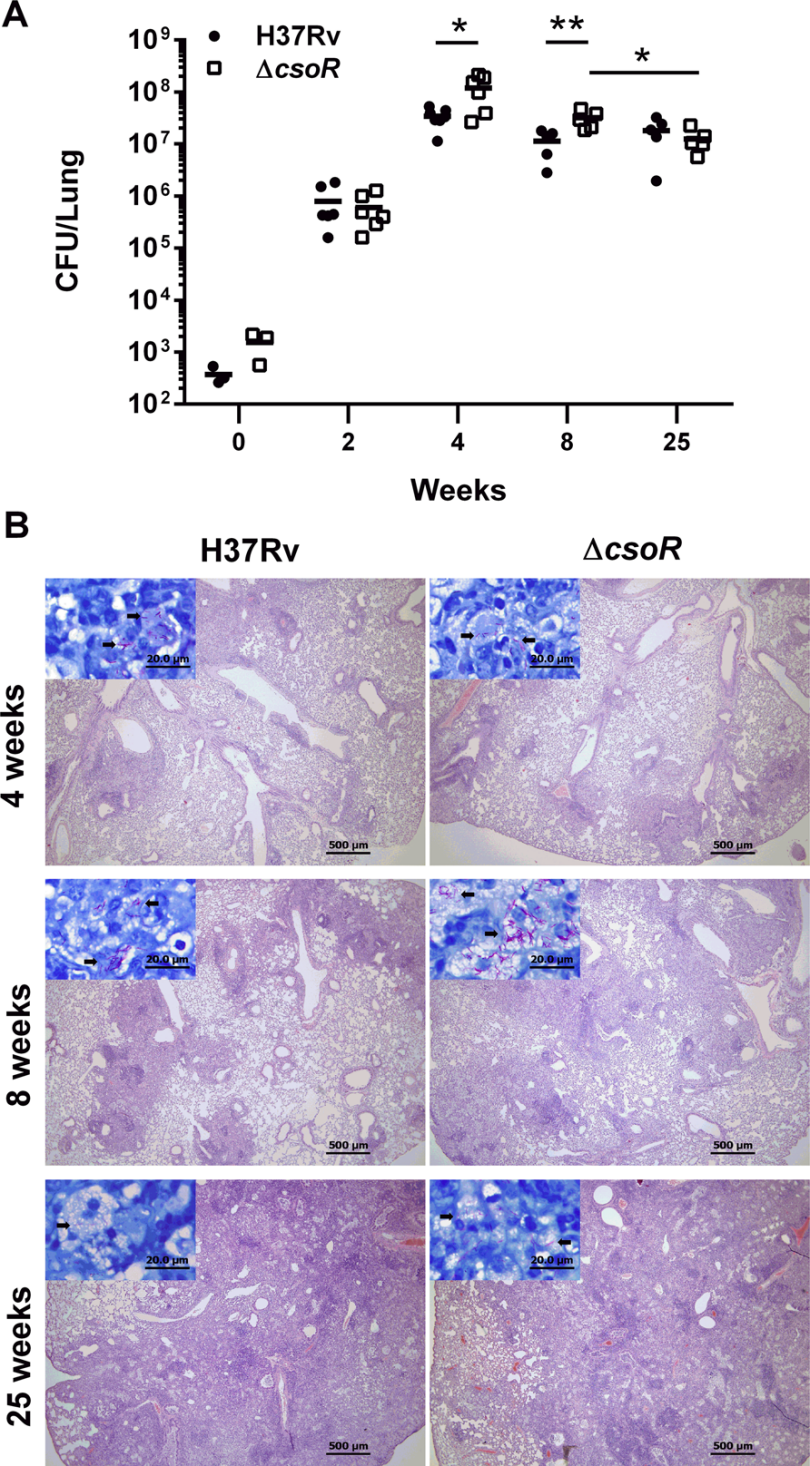
Growth of Δ*csoR* in the lungs during mouse infection. **(A)** Groups of BALB/c mice were infected by aerosol route with either *M*. *tuberculosis* H37Rv (filled circles) or Δ*csoR* (open squares). Shown are CFU per lung for each individual mouse over the course of 25 weeks across two independent experiments. Asterisks indicate significance with the following *P*-values: * *P* < 0.015; ** *P* < 0.005. **(B)** Histopathology for mice infected with H37Rv or Δ*csoR* at 4 and 8 weeks. Mouse lung sections were stained with H&E and are shown at 40× magnification (scale bar = 500μm). Insets show Ziehl-Neelson stained sections of lung tissue with pink bacilli indicated by black arrows at 1000× magnification (scale bar = 20μm).

To gain a broader understanding of the role of *csoR* during *M*. *tuberculosis* infection, BALB/c mice were infected with H37Rv wild type or Δ*csoR* by aerosol infection. The groups infected with wild type received on average 370 CFU per animal while Δ*csoR* infected mice received closer to 1500 CFU per animal; however this difference in inoculum did not reach significance. In fact, by week 2 Δ*csoR* showed on average slightly less colonization than wild type. Despite this early trend, by weeks 4 and 8 Δ*csoR* showed a significant increase in survival compared to wild type (*P* = 0.015 and *P* = 0.005 respectively). This increase, however, was not sustained long term, as by week 25 no significant difference in survival between wild type and knockout was apparent (Fig 3A). While the colonization of the wild type strain was consistent from week 8 to week 25, Δ*csoR* showed a significant drop from week 8 to week 25 (*P* = 0.007), suggesting that the improved growth rate during early chronic stage was not sustainable throughout the duration of chronic infection. Unlike the lungs, no significant difference in survival was seen between wild type and Δ*csoR* when spleen and liver were assayed, although mean CFU/g were consistently higher in the mutant strain in both organs (S3 Fig). Body weight of the mice did not vary significantly between groups after week 2 of the experiment, when mice infected with ΔcsoR were on average 1.2g lighter than those infected with wild type (P = 0.005) (S3 Fig). Histopathology of the lungs, spleen, and liver (Fig 3B) was scored by a trained pathologist. For both strains, lymphocytic infiltration and granulomatous reaction in the lungs worsened as the infection progressed, while minimal pathology was seen in the spleen and liver. No significant difference was noted between strains at any time point for any of the tissues (S4 Fig). In sum, *csoR* conferred a moderate disadvantage to wild type *M*. *tuberculosis* during the early weeks of murine infection, but may be required for long term survival.

### The global transcriptional response of *M*. *tuberculosis* Δ*csoR*

**Fig 4:**
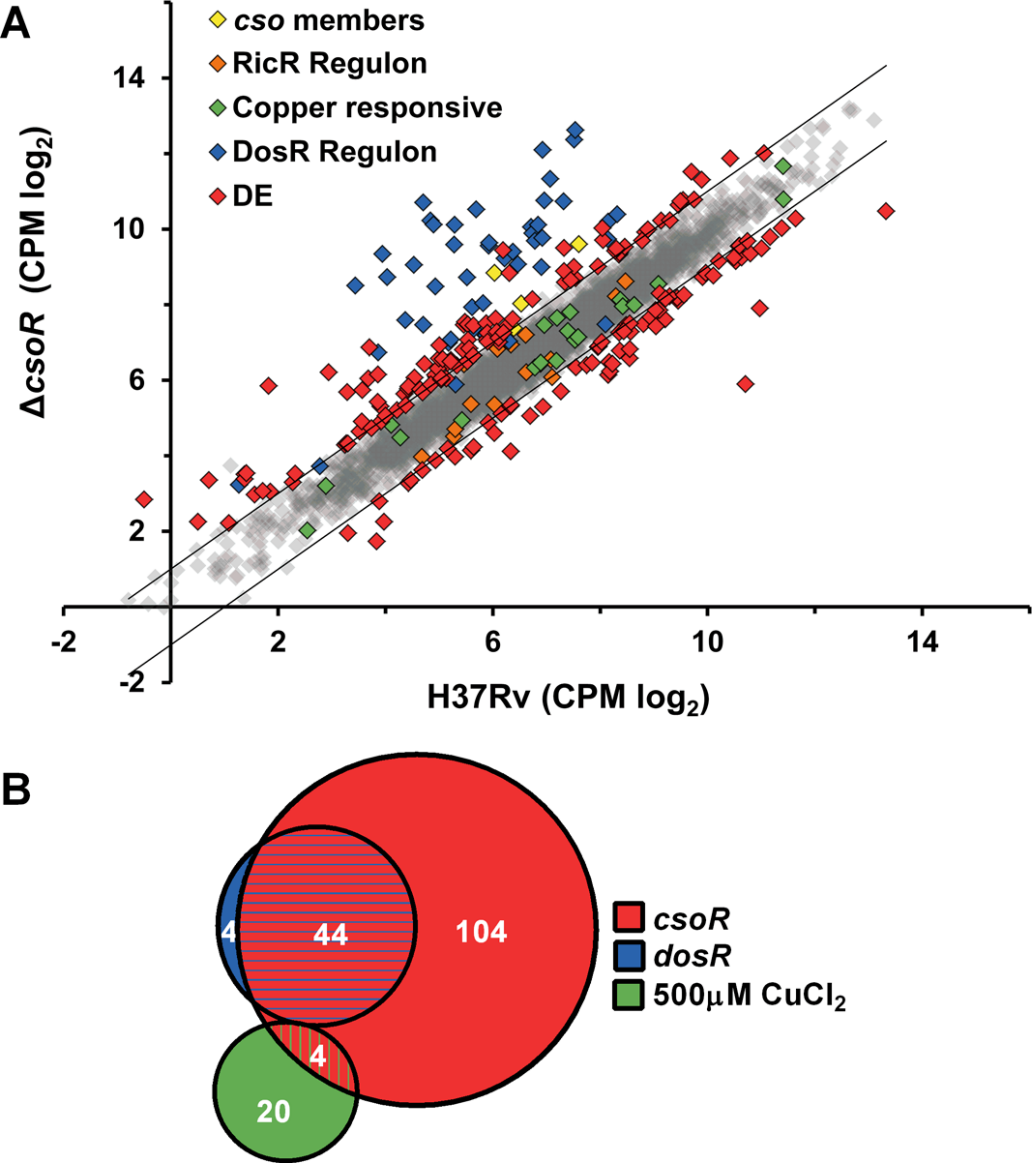
Analysis of the Δ*csoR* transcriptome. **(A)** Counts per million (CPM) of each gene detected in our RNA-Seq study, plotted for *M*. *tuberculosis* Δ*csoR* versus H37Rv wild type. Highlighted are members of the *cso* (yellow), members of the RicR regulon (orange), genes responsive to copper, but not part of the *cso* or RicR regulon (green), members of the DosR regulon (blue), and significantly differentially expressed genes as determined by an FDR ≥ 0.05 not included in the above groups (red). Two parallel, black lines demarcate the region outside of which differential expression values exceed our cutoff of a 2.0 fold difference between strains. Genes not meeting both cutoff values are shown as semi-transparent grey diamonds. Data points show the mean of two biological replicates. **(B)** Overlap of the induced *csoR* regulon (red) with 500 CuCl_2_ inducible genes (green) and genes induced under the control of *dosR* (blue). Of the 152 genes induced in the Δ*csoR* strain compared to H37Rv wild type, only 4 overlapped with the 24 genes induced in H37Rv wild type when exposed to copper stress at 500μM CuCl_2_. DosR inducible genes showed substantial overlap with Δ*csoR* with 44 out of 48 overlapping. The diagram is area-proportional.

To better understand the breadth of influence *csoR* has directly as a regulator, and indirectly over copper homeostasis within the cell, the Δ*csoR* transcriptome was interrogated and compared to that of wild type *M*. *tuberculosis* H37Rv using RNA-Seq analysis. Our previous work [13] and that concerning *csoR* in other species such as *B*. *subtilis* [20] has suggested that under copper stress CsoR derepresses its regulon, and therefore few transcriptional differences between wild type and *csoR* knockout strains would be noted when compared under copper stress. Thus, we compared the transcriptomes of both wild type *M*. *tuberculosis* and Δ*csoR* grown in copper-free Sauton’s media. Details of the number of reads and how they mapped can be found in S1 Table. Differential expression analysis using strict cutoffs requiring differentially expressed genes to have a fold change ≥ |2.0| and a false discovery rate (FDR) of ≤ 0.05 left us with 223 genes that were significantly, differentially expressed between the mutant and wild type strains (S2 Table). Of those genes 152 were induced, while 71 were repressed in Δ*csoR* compared to wild type (Fig 4A). To confirm these results, 10 genes were selected– 6 induced and 4 repressed–for quantitative, real-time PCR (qRT-PCR). All 10 were confirmed to be induced or repressed in the same direction, and with similar magnitude as the RNA-Seq data (S5 Fig).

It has already been established by our group and others that *csoR* represses its own operon in the absence of copper [13, 21]. Therefore, we first looked at the expression levels of the *cso*. As expected under low levels of copper, the *cso* was moderately induced in the Δ*csoR* strain as compared to wild type (Fig 4A; Table 1). This included the observed induction of *csoR* itself as sufficient remnants of the mutated *csoR* gene remained for successful RNA-Seq analysis. As CsoR is an established copper-responsive regulator, the list of differentially expressed genes in Δ*csoR* compared to wild type was interrogated for the 30 genes known to be responsive to copper in *M*. *tuberculosis* as determined by previous microarray analysis [6]. Very few genes overlapped; in fact, the only overlapping gene outside of the *cso*, Rv0848, was repressed in the Δ*csoR* strain despite being induced under copper stress (Fig 4A and 4B; Table 1). These results suggest that *csoR* may indeed only directly control expression of its own operon as previously suggested by Festa, *et al* [21]. While no significant changes in the majority of copper responsive genes were noted, most outside the *cso* (n = 18) were down regulated, short of our cutoff values. These include another copper sensing regulator, *ricR* (-1.5, FDR = 0.18). Among the RicR regulon [21], two genes, Rv0847 and Rv0848, were significantly repressed. Additionally, several of the other genes under the control of RicR (n = 7), including *mymT* a copper binding metallothionein [22] and *mmcO*, a multicopper oxidase which is exported across the cytoplasmic membrane [23], were slightly, though not significantly down regulated (Fig 4A), and this trend was also confirmed by qRT-PCR. These results clearly demonstrate that the CsoR and RicR regulons are distinct; however deregulation of the CsoR regulon may contribute to a slight, perhaps compensatory, increase in repression of the RicR regulon.

**Table 1:** Selected genes differentially expressed in *M*. *tuberculosis* Δ*csoR* as compared to wild type.

Locus	Name	Description	Fold Change^a^	FDR^b^
***Copper Responsive Genes***				
Rv0848	*cysK2*	cysteine synthase A	-2.23	4.79E-04
Rv0967	*csoR*	copper-sensing transcriptional repressor	6.43	9.11E-22
Rv0968	* *	conserved hypothetical protein	2.92	2.61E-09
Rv0969	*ctpV*	metal cation transporting P-type ATPase	4.11	3.51E-19
Rv0970	* *	conserved membrane protein	1.96	8.89E-05
***Transcriptional Regulators***				
Rv0081	* *	transcriptional regulator	6.99	2.46E-28
Rv0144	* *	transcriptional regulator, *tetR*-family	-2.28	2.67E-06
Rv0386	* *	transcriptional regulator, *luxR*/*uhpA*-family	2.42	4.60E-07
Rv0452	* *	transcriptional regulator	2.31	1.30E-05
Rv0981	*mprA*	mycobacterial persistence regulator	-2.16	4.05E-06
Rv1129c	* *	transcriptional regulator	-7.85	1.53E-07
Rv1657	*argR*	arginine repressor argR	-2.83	7.33E-09
Rv1985c	* *	transcriptional regulator, *lysR*-family	-1.98	5.34E-04
Rv2017	* *	transcriptional regulator	2.19	3.97E-06
Rv2621c	* *	transcriptional regulator	2.59	2.23E-04
Rv2779c	* *	transcriptional regulator, *lrp*/*asnC*-family	-3.02	8.11E-12
Rv3132c	*dosS*	two component system sensor histidine kinase	4.38	2.74E-22
Rv3133c	*dosR*	two component system transcriptional regulator	7.69	9.09E-37
Rv3197A	*whiB7*	transcriptional regulator whiB-like	-2.00	3.50E-04
Rv3334	* *	transcriptional regulator, *merR*-family	2.94	4.37E-10
Rv3855	*ethR*	tetR-family transcriptional repressor	2.09	1.34E-03
***INH Responsive Genes***				
Rv1592c	* *	conserved hypothetical protein	4.51	4.63E-23
Rv1854c	*ndh*	NADH dehydrogenase	2.09	2.27E-04
Rv2243	*fabD*	malonyl CoA-acyl carrier protein transacylase	3.42	4.10E-12
Rv2245	*kasA*	3-oxoacyl-[acyl-carrier protein] synthase 1	2.96	2.92E-09
Rv2247	*accD6*	acetyl/propionyl-CoA carboxylase beta subunit	2.16	4.42E-05
Rv2482	*plsB2*	glycerol-3-phosphate acyltransferase	-2.08	5.08E-06
Rv2846c	*efpA*	membrane efflux protein	2.35	8.54E-06

^a^ Fold change in the ΔcsoR transcriptome as compared to wild type.

^b^ As determined by Benjamini and Hochberg's algorithm

With evidence that CsoR directly regulates only its own operon, it seemed likely that other forms of regulation were at play in the Δ*csoR* strain. While no sigma factors were found to be differentially expressed, numerous transcriptional regulators were among those differentially expressed (n = 16) (Table 1). Therefore, the genes under transcriptional control of these regulators may represent a significant portion of the differentially expressed genes in our dataset. One striking pattern was the induction of 44 out of 48 members of the *dosR* regulon, known for its role in response to hypoxia, nitric oxide, and dormancy [24, 25] (Fig 4B). These members of the *dosR* regulon were among the most highly induced genes in our dataset (Fig 4A). Another notable regulator involved in broad regulation of genes throughout various stages of hypoxia, Rv0081, was also induced [26]. This again suggests that *csoR* itself may not be directly controlling the expression of these genes, but rather derepression of its own regulon may be creating hypoxia-like stress conditions to which *dosR*, Rv0081, and other transcriptional regulators respond. Overall, our analyses suggest that CsoR represses its own operon, and absence of *csoR* may lead to a disruption in copper homeostasis, leading to a hypoxia or NO type stress response.

### The impact of CsoR on drug resistance

During analysis of the *csoR* impacted genes it was noted that genes involved in the response to isoniazid (INH) were enriched, with 7 of the 23 known responders being induced. These 7 genes included several genes involved in fatty acid synthesis as well as membrane efflux protein, *efpA* (Table 1). Based on this finding and a previous study that showed the absence of *M*. *smegmatis* copper-responsive regulator GfcR increased INH resistance [27], we decided to investigate this phenotype in our mutant strain. Disc and MABA assays [19] examining inhibition of *M*. *tuberculosis* H37Rv and *ΔcsoR* growth revealed a slight but not significant difference in INH susceptibility between strains (S6 Fig). It may be that the induced INH responsive genes are responsive to more general stress conditions, and therefore are not those most relevant for mitigating INH related stress.

### Expression dynamics of the copper sensing operon in the absence of *csoR* during copper stress

**Fig 5:**
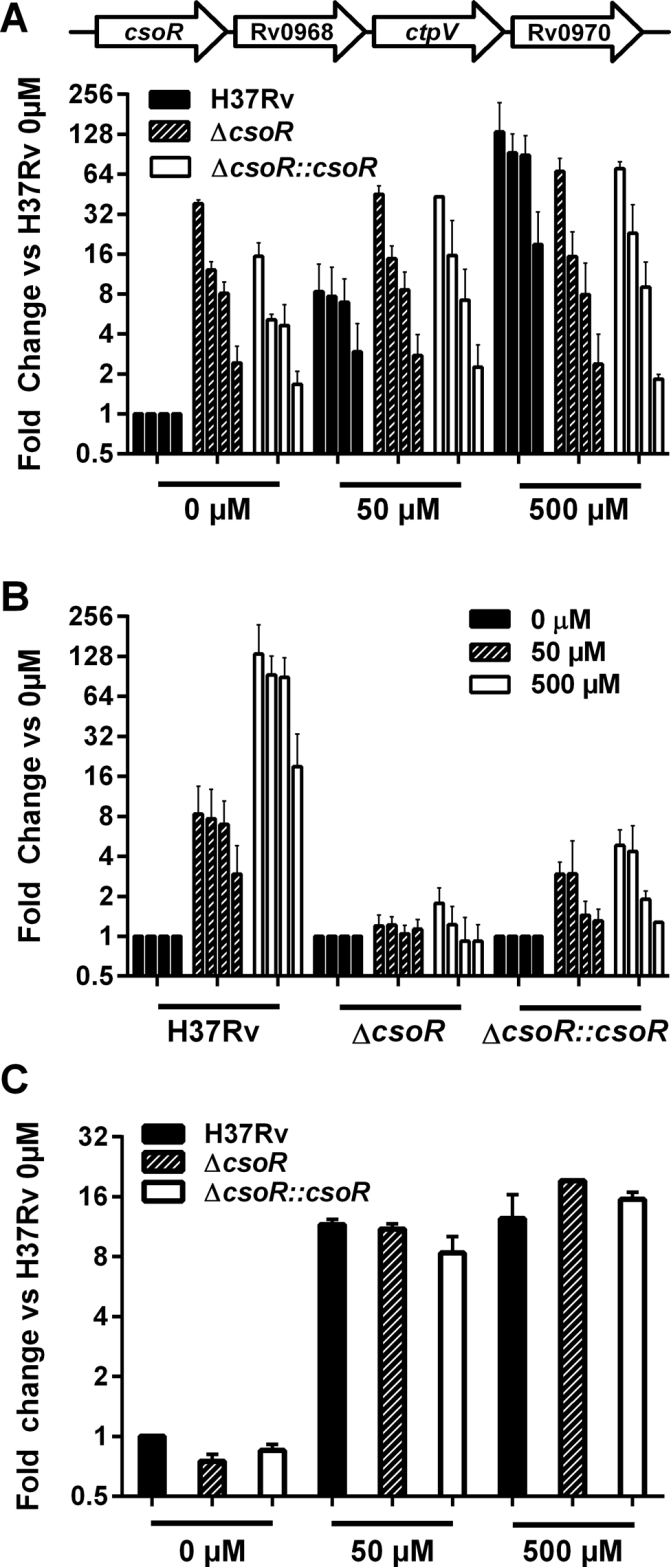
Expression of *cso* genes during copper stress in the absence of *csoR*. qRT-PCR was used to analyze expression levels of *csoR*, Rv0968, *ctpV*, and Rv0970. A schematic of the operon (not drawn to scale) is shown above the graph and the order of the genes in the operon corresponds to the order in which expression levels are graphed for each gene. **(A)** *M*. *tuberculosis* strains H37Rv (black), Δ*csoR* (striped), or Δ*csoR*::*csoR* (white) were exposed to CuCl_2_ at 50 or 500μM or left untreated (0μM) for 3 hours. Values shown are the mean fold change between each gene and its untreated wild type counterpart after normalization to *sigA* expression levels. **(B)** A second comparison of the same samples was done showing the mean fold change between each gene after normalization to *sigA* expression levels at 50μM (striped) or 500μM (white) and its untreated counterpart (black) from the same strain. Data represent one of two similar biological replicates. Error bars represent the standard error of the mean from three technical replicates. **(C)** Expression levels of *mmcO* as determined by qRT-PCR analysis of the same samples–H37Rv (black), Δ*csoR* (striped), or Δ*csoR*::*csoR* (white)–left untreated (0μM) or stressed with 50μM or 500μM CuCl_2_. Fold change is shown as expression levels of each gene relative to expression levels in untreated wild type culture after normalization to *sigA* expression levels. Data represent one of two similar biological replicates. Error bars represent the standard error of the mean from two technical replicates.

While we have shown that the *cso* is induced in the absence of *csoR* and copper stress, it is still unclear if CsoR deregulation alone accounts for the induction of the *cso* under copper stress. To better examine the dynamics of CsoR regulation of the *cso* during copper stress, we utilized qRT-PCR to profile the expression levels of *csoR*, Rv0968, *ctpV*, and Rv0970. Samples for analysis were taken from *M*. *tuberculosis* H37Rv, *ΔcsoR*, and *ΔcsoR*::*csoR* left untreated or exposed to either 50 or 500μM CuCl_2_ for 3 hours. As expected, qRT-PCR analysis of all 4 *cso* genes showed induction of the operon in the absence of *csoR* (Fig 5A). This level of induction was on par with the induction of the operon under moderate copper stress (50μM CuCl_2_) in the wild type strain, but was less than the expression levels seen in wild type under high copper stress (500μM CuCl_2_). Expression levels of the complement strain were similar to that of the knockout. Interestingly, as the level of copper stress increased for the *ΔcsoR* strain, no change in *cso* expression was seen at 50 or 500μM CuCl_2_ compared to untreated *ΔcsoR*, unlike in wild type (Fig 5B). Analysis of *ΔcsoR*::*csoR* on the other hand did reveal a modest rescue of the copper responsive phenotype.

To test the hypothesis that the *cso* may be under the control of multiple copper sensitive regulators and may not be responding to copper stress in *ΔcsoR* due to increased copper export, we sought to investigate the levels of copper present within each strain. Direct measures of copper such as neutron activation analysis are not reliably precise enough to detect small differences in copper ions within *M*. *tuberculosis* [15, 21]. Therefore *mmcO*, part of the copper responsive RicR regulon, was picked as an indirect indicator of copper stress experienced by the cell [18]. Thus, samples of wild type, Δ*csoR*, and complement strains under different levels of copper stress were examined by qRT-PCR analysis for *mmcO*. No significant difference in *mmcO* induction was detected between strains (Fig 5C). As with the global transcriptome analysis, *mmcO* was slightly down-regulated in *ΔcsoR* as compared to wild type in copper-free media. This could be indicative of reduced levels of copper present in the *M*. *tuberculosis* cytoplasm in *ΔcsoR* in the absence of copper stress. However, once copper was added, *mmcO*, unlike the *cso*, increased drastically in the mutant strain. A similar profile was also observed when copper responsive gene *mymT* was analyzed (S7 Fig). These results indicated that under copper stress, copper levels between the three strains were equivalent, excluding the possibility that differences in intracellular copper were contributing to the differential expression of the *cso*. We cannot, however, rule out the possibility that other regulators, responsive to copper or related stress, participate in the regulation of the *cso*.

## Discussion

Recently, great interest has been taken in the importance of copper homeostasis and its role in bacterial pathogenesis and host defense [28]. Previously, our group elucidated the regulatory mechanisms behind the copper responsive transcriptional repressor CsoR [13]. We have also shown the shuttling activity of one of its directly regulated genes, *ctpV*, in response to copper stress [15]. In this study, we examined the importance of this negative regulator, CsoR, to both copper and host-mediated stress in a murine model of tuberculosis. While a multifaceted role of CsoR had been previously suggested—in which CsoR has a buffering effect preventing damage from free copper [13]—our results suggest that the genes within the CsoR regulon, rather than CsoR itself, are primarily responsible for directly mitigating the harmful effects of copper stress. Consistent with this hypothesis, the survival curves of Δ*csoR* at 500μm CuCl_2_ are reminiscent of those seen for *M*. *tuberculosis* Δ*ctpV*::*ctpV*, which overexpresses *ctpV*, a copper exporting member of the *csoR* regulon [15]. Less expected was the dramatic difference in strain survival depending on the use of a fresh, log stage inoculum or one that had been grown to stationary phase before inoculation in copper containing media. One possible cause of this difference could be a slower response to copper stress in the stationary phase cultures, exaggerating the otherwise slight copper resistant phenotype of Δ*csoR*. On the other hand, it may be that the *cso* has a more important role to play during dormancy than during logarithmic phase growth, particularly after lengthy exposures to copper that may allow copper to build up within the cell.

The role of the *cso* in dormancy is supported by the observed induction of *csoR* throughout the enduring hypoxic response [29]. An increased, hypoxia-specific copper response may be physiologically important as not only is copper more toxic under hypoxic conditions [30], but hypoxia stimulates macrophages to increase copper uptake [31]. In fact hypoxia and high copper levels have both been found within granulomas of guinea pigs during *M*. *tuberculosis* infection, emphasizing the need to adapt to combat both stress conditions simultaneously [4, 32]. At the same time, the *M*. *tuberculosis* requirement for copper under hypoxia is decreased as copper-independent cytochrome *bd* oxidase is induced, reducing the need for copper-dependent cytochrome *c* oxidase [12]. The relationship of *csoR*, copper stress, and hypoxia-induced dormancy is currently being investigated further.

As the host environment [32], particularly the mycobacterial phagosome [5] is known to harbor high levels of copper during *M*. *tuberculosis* infection as a potential antimicrobial defense mechanism, we were interested in testing if the increased resistance to copper of the Δ*csoR* strain translated to our murine aerosol infection model. Mouse infections revealed that despite similar CFU counts at week 2, the mutant strain multiplied and survived nearly a log fold above wild type through the early stages of chronic infection at weeks 4 and 8. These results might be attributed to the preparedness of the Δ*csoR* strain to face host stress conditions even before they were detected; as transcriptional analysis indicated, the mutant strain already had genes induced that typically respond specifically to host stressors such as copper (*ctpV*), nitric oxide, and hypoxia (*dosR* regulon). Toxic copper levels and nitric oxide are stressors faced by *M*. *tuberculosis* within the phagosome of macrophages, especially those that have been activated. This activation follows the initiation of the adaptive immune response to *M*. *tuberculosis* infection taking place around weeks 2 to 4 [33], after which the Δ*csoR* strain shows improved survival over wild type. Previously published data examining our Δ*cso* strain demonstrated a phenotype opposite of that observed for Δ*csoR* during mouse infection [34]. Unlike our Δ*csoR* strain, where the remaining members of the *cso* are induced, the Δ*cso* strain was attenuated, particularly during the later time points of chronic infection [34]. This earlier study, and our transcriptional study showing the induction of the cso during mouse infection [35], highlight the importance of the whole *cso* operon to the survival of *M*. *tuberculosis* during infection.

To better understand the control CsoR has over its own operon, we studied transcriptional expression of the *cso* in wild type, knockout, and complement strains in untreated culture and under two levels of copper (50, and 500μM). As expected, we found that without *csoR*, the *cso* genes were induced in the absence of copper. Notably, this induction is not to the same level as the induction seen in wild type under high levels of copper stress (500μM). This indicates that at high levels of copper, CsoR derepression may not be solely responsible for *cso* induction. This hypothesis is consistent with the idea that genes outside of the core copper responsive regulon (n = 15 induced at 50μM CuCl_2_) are induced to address secondary stress such as the presence of reactive oxygen and nitrogen species at higher copper levels (n = 30 induced at 500μM CuCl_2_) [6]. It could be that a second transcriptional regulator is required to further induce the *cso* in response to this toxic level of copper stress. Therefore, disruption of sequence near the binding site of a second regulator may have contributed to a lesser level of *cso* induction. One such candidate may be transcriptional regulator Rv2324 predicted to bind within *csoR* just upstream of the deletion site [26]. Therefore, the attenuated *cso* induction under high levels of copper stress may have attributed to a more modest survival phenotype of the Δ*csoR* strain under copper and host stress than might otherwise be expected.

Despite the apparent advantage of losing *csoR* under both copper and host stress, our global transcriptional analysis reveals the important role *csoR* plays in the cell under normal growth conditions. While this work has not ventured to define the direct targets of CsoR regulation, our results support previous findings that CsoR may only regulate its own operon [21]. Both studies found the other members of the *cso* to be induced in the *csoR* mutants to similar levels. The previous study [21], however, found no differentially expressed genes outside of the *cso*, in stark contrast to our list of 223 differentially regulated genes. It is possible, as the authors point out, that the previously studied strain is a polar knockout, unlike ours. It may be that translation of the *cso* in their Δ*csoR*:: *hyg*_*R*_ strain was not as efficient as in the Δ*csoR* strain discussed here, therefore mitigating any stress we posit is placed on the cell when the operon is deregulated in the absence of *csoR*. We hypothesize that this stress is derived from the consequences of *cso* overexpression in the absence of copper. Interestingly, outside of the *cso*, the only copper responsive genes to be differentially expressed were down regulated [6]. It may be that lower levels of copper in the Δ*csoR* strain, due to increased Cu export by CtpV, tightened copper mediated repression of these genes such as *mmcO* and *mymT*, by regulators such as RicR. This tightening of repression was observed in our RNA-Seq and confirmed by qRT-PCR above. This compensatory response suggests a hierarchy of copper regulation in *M*. *tuberculosis* with CsoR having a key role. It should also be noted, however, that these previous studies were conducted using microarray analysis, which can be less sensitive to differential expression than RNA-Seq analysis [36]. Therefore, the absence of more extensive overlap among these studies may be partially due to the difference in technique used.

**Fig 6:**
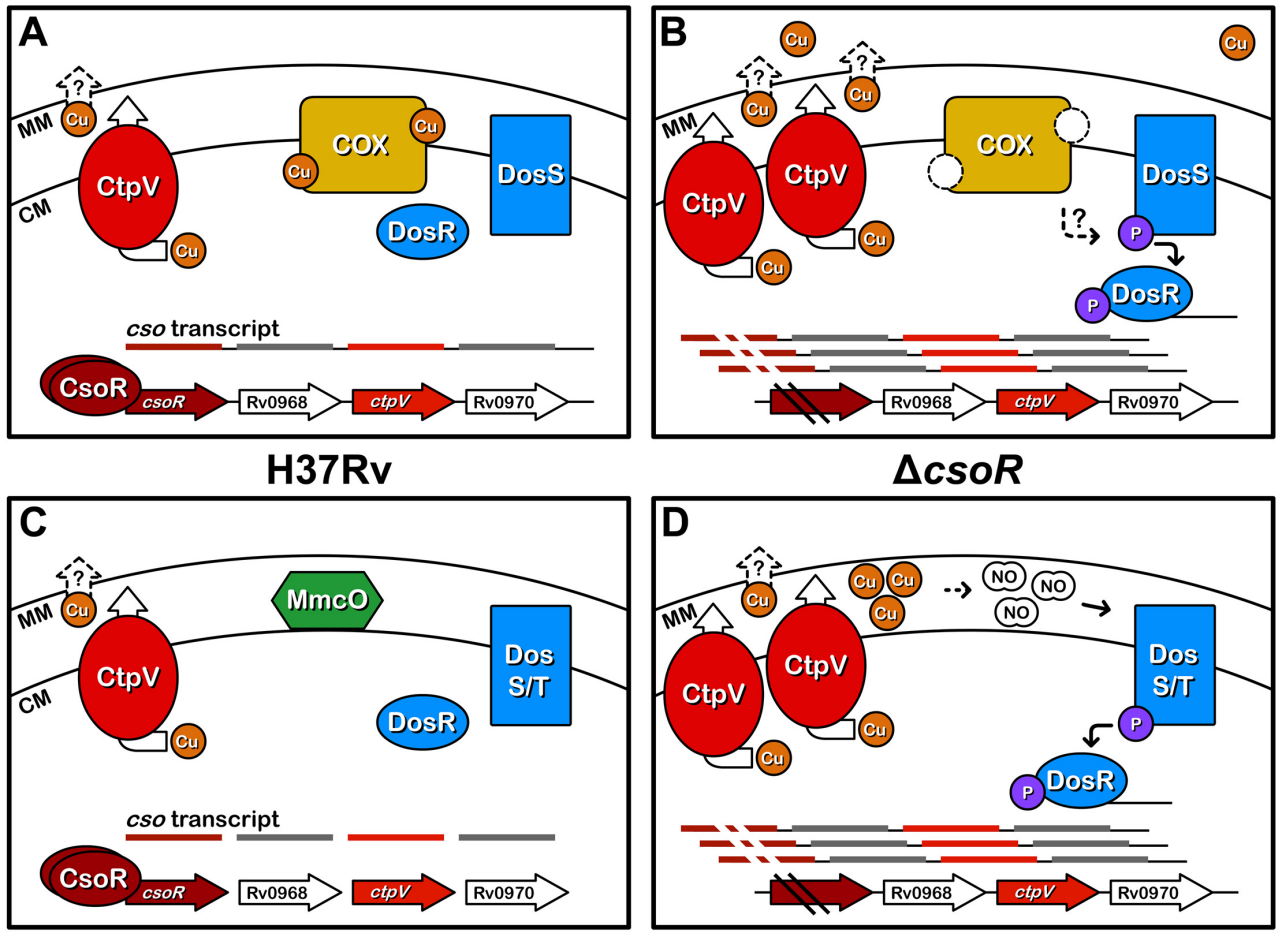
Potential consequences of Δ*csoR* disrupted copper homeostasis. Overexpression of the *cso* and the proteins it codes for after deletion of *csoR* (dark red) may result in excessive levels of copper export by CtpV (red). **(A)** As a result, pools of copper (orange) for metalloenzyme usage may be depleted, reducing the levels of functional proteins such as cytochrome c oxidase (yellow), as seen in the panel on the right. This could potentially slow the electron transport chain leading to the induction of the DosR (blue) regulon through the redox sensor, DosS (blue) [38]. The wild type strain is shown in the panel on the left for comparison. **(B)** Alternatively, the increased cytoplasmic export of copper may lead to a buildup of copper in the mycobacterial periplasmic space where MmcO (green) usually assists in copper detoxification. The increased copper stress in this region may be magnified, however, by the down regulation of *mmcO* in the mutant strain. Free copper can lead to the production of reactive nitrogen species, such as NO [39]. NO can trigger expression of the DosR regulon [25]. The wild type strain is shown in the panel on the left for comparison. CM; cytoplasmic membrane. MM; mycomembrane. Cu; copper. NO; nitric oxide. COX; cytochrome c oxidase. P; phosphoryl group.

A major consequence of the loss of *csoR* and the deregulation of the *cso* was the induction of the DosR regulon. While culture manipulation can cause a slight but significant induction of the DosR regulon [37], great care was taken to quickly process all samples to be used for RNA analysis in an identical fashion. The observed induction of the DosR regulon was substantial, and likely too high to be caused by handling differences. While the mechanism for this induction remains unresolved, we have modeled two opposing hypotheses (Fig 6). The first is based on data suggesting that disruption in the electron transport system or cytochrome c oxidase induces expression of the DosR regulon [25, 38] (Fig 6A). As cytochrome c oxidase, involved in the terminal steps of cellular respiration, is a copper metalloenzyme, *cso* induction resulting in excessive copper export could negatively impact its function. This block in respiration may contribute to the activation of the DosR regulon.

A second possibility could be that NO is directly inducing the DosR regulon in our Δ*csoR* strain (Fig 6B). While we hypothesize that copper may be present at lower levels in the cytoplasmic space, CtpV is thought to export copper across only the cell membrane. We do not know if expression levels of genes involved in the export of copper across the mycobacterial cell wall were affected, as the only gene proposed to fulfill this role in *M*. *tuberculosis*, Rv1698, was later excluded as a potential candidate [32, 40]. Thus it is possible that copper could be accumulating and causing damage in the mycobacterial periplasmic space of Δ*csoR*. This damage could be exacerbated by the slight down-regulation of a recently characterized periplasmic multicopper oxidase, *mmcO* [23]. Cu(I) can generate NO through interactions with S-nitrosothiols [39], which could subsequently induce the DosR regulon.

These scenarios and the stressed transcriptional profile of the Δ*csoR* strain highlight the potential harm that can be done even at low levels of copper and emphasize the need for balance in regulating the level of intracellular copper, as aided by *csoR*. This increased understanding of the balance of copper *M*. *tuberculosis* must maintain during infection may ultimately help guide development of antimycobacterial therapies that wish to utilize copper or target the copper stress response. Overall this study has revealed new insights into the importance of copper regulation and *csoR*, not only under copper stress, but also in its absence. Similar copper regulators are present in other intracellular pathogens and similar scenarios could be at play in these organisms as well.

## Materials and Methods

### Strain construction

Strains used in this work include *Mycobacterium tuberculosis* H37Rv, and its derivatives *M*. *tuberculosis* Δ*csoR* and *M*. *tuberculosis* Δ*csoR*::*csoR*. To create an inactivating mutation in *csoR* in *M*. *tuberculosis*, we first generated a Δ*csoR*::*hyg*_*R*_ strain using specialized transduction and homologous recombination as outlined previously [14, 34] and confirmed by sequencing and Southern blot [34]. To determine the polar or nonpolar nature of the construct, RNA was isolated from the strain using a TRIzol® Reagent based method before treatment with TURBO™ DNase (Ambion®, Austin, TX) until PCR negative. RNA was then reverse transcribed to cDNA using Superscript® III (Invitrogen, Carlsbad, CA). cDNA was then screened by PCR for the expression of the genes downstream of *csoR*: Rv0968, *ctpV*, and Rv0970. Our analysis indicated that the mutant was polar.

To construct an unmarked mutant strain, a helper plasmid, pYUB870, was used to remove the *hyg*_*R*_ gene as described previously [14], leaving behind a stop codon at the 14^th^ amino acid of the *csoR* gene. Again these results were confirmed by sequencing and the nonpolar nature of this mutant was demonstrated as described above. This strain was used for the construction of a complement strain. The *csoR* gene and the 200bp promoter region upstream of the *csoR* transcriptional start site were cloned into the integrative, *E*. *coli*-mycobacterial shuttle vector pMV306, originally derived from pMV361 [41]. The vector, containing a kanamycin resistance marker, was electroporated into *M*. *tuberculosis* Δ*csoR*. Successful electroporants were identified by screening colonies growing on Middlebrook 7H10 media supplemented with 10% ADC and 30 μg/ml kanamycin by PCR targeting *csoR* and the upstream portion of the pMV306 vector. The final clone was verified by sequencing.

### Growth conditions

Starter cultures for experiments were grown from frozen stocks in liquid media to late log stage (~OD_600_ 1.0) in Middlebrook 7H9 (Remel™, Lenexa, KS) with 10% ADC and 0.05% TWEEN 80. For all experiments, 30μg/ml kanamycin were included in growth media used for *M*. *tuberculosis* Δ*csoR*::*csoR*. Before use in downstream experiments, bacteria taken from 7H9 cultures were washed twice with copper-free Sauton’s media. Similarly, all reagents and media used in copper sensitive experiments were treated with 0.2% (w/v) Chelex® 100 (Sigma-Aldrich, St. Louis, MO) to remove traces of copper prior to addition of other metal supplements before use. All glassware was acid washed with 1M nitric acid to remove any traces of metal ions. For transcriptional experiments, washed *M*. *tuberculosis* strains H37Rv, Δ*csoR*, and Δ*csoR*::*csoR* were inoculated to an OD_600_ of 0.10 in 30 or 100 ml Sauton’s media containing 0.05% TWEEN 80. At late log phase (~OD_600_ 1.0) cultures were exposed to untreated media or 50 or 500μM CuCl_2_ for 3 hours before collecting cultures for RNA extraction as detailed previously [6].

### Microplate Alamar Blue Assay (MABA)

To quantify the level of resistance of *M*. *tuberculosis* constructs to copper, MABA assays were performed as described previously [18, 19] with a few modifications. Briefly, the outer wells of flat bottom 96-well microplates were filled with sterile ddH_2_O to prevent evaporation. Inner wells were filled with 100ul Sauton’s broth containing serial dilutions of CuCl_2_ in duplicate or no added CuCl_2_ as a control and *M*. *tuberculosis* strains H37Rv, Δ*csoR*, and Δ*csoR*::*csoR* inoculated to a theoretical OD_600_ of 0.05. A series of nine, two-fold dilutions were made with CuCl_2_ from 16 to 4000μM. On day 5 after inoculation, 30μl Alamar Blue (Invitrogen; Carlsbad, CA): TWEEN 80 (1:1) were added to a control well and incubated an additional 24hrs. When a shift in color from blue to pink was seen on the following day, 30μl Alamar blue:TWEEN 80 (1:1) was added to each of the remaining wells. Data were collected the following day and the blue wells containing the lowest concentration of CuCl_2_ were recorded for each series and each strain as the minimum inhibitory concentration (MIC).

### Mouse infections

BALB/c mice (Harlan, Indianapolis, IN) were infected with *M*. *tuberculosis* H37Rv wild type or Δ*csoR* strains using an aerosol infection model with the Glas-Col® Inhalation Exposure System (Glas-Col, LLC, Terre Haute, IN) as outlined before [15]. At 2, 4, 8, and 25 weeks groups of 3 to 5 mice were euthanized by cervical dislocation following anesthetization by isoflurane, and lung, spleen, and liver were collected for colony counts and histopathology [15]. Aside from the 25 week time point, the experiment was completed in duplicate.

### Ethics statement

Animal experiments were approved by the Institutional Animal Care and Use Committee, University of Wisconsin-Madison (V1095) in compliance with the regulations set in place by the Public Health Service Policy on the Humane Care and Use of Laboratory Animals, overseen by the NIH Office of Laboratory Animal Welfare (OLAW). Mice were monitored daily by caretakers for signs of illness or distress and all efforts were taken to prevent animal suffering. No unexpected deaths occurred during the study and all mice reached predetermined endpoints without showing signs such as behavioral changes, fur ruffling, anorexia, or lethargy that would require the use of a humane endpoint.

### Quantitative, real-time PCR

RNA was isolated for qRT-PCR immediately after collecting the cultures using a TRIzol based method described previously [15, 34]. RNA was treated with TURBO DNase until PCR negative to remove contaminating DNA. For qRT-PCR analysis of the *cso*, 1–2μg RNA were used as template for cDNA using Superscript III. A SYBR green based qRT-PCR protocol utilizing GoTaq® qPCR Master Mix (Promega, Madison, WI) and the StepOnePlus™ Real-Time PCR System (Applied Biosystems®, Foster City, CA) were used. Expression of all genes was normalized to *sigA* expression levels. Analysis was carried out using LinRegPCR [42]. Two biological replicates with no less than two technical replicates each were completed.

### Transcriptional profiling through RNA sequencing

For transcriptional profiling with RNA-Seq technology, RNA was isolated from cultures grown in Sauton’s broth to late log phase and treated with TURBO DNAse as done with samples for qRT-PCR. RNA integrity was confirmed using the 2100 Bioanalyzer (Agilent, Santa Clara, CA). The 23S, 16S, and 5S rRNAs were depleted from the samples using the Ribo-Zero™ Magnetic Kit for bacteria (Epicentre, Madison, WI). Biological replicates of prepared rRNA-reduced RNA were submitted to the University of Wisconsin Biotechnology Center’s DNA Sequencing Facility where they were prepared for sequencing using the TruSeq RNA Sample Preparation Kit (Illumina®, San Diego, CA). The samples were run on an Illumina HiSeq™ 2000 to generate 100bp single reads. Base calling was done in CASAVA 1.8.2. Before differential expression analysis the quality of the FASTQ files was checked using the FASTX-Toolkit developed in the lab of Gregory Hannon at Cold Spring Harbor (http://hannonlab.cshl.edu/fastx_toolkit/index.html). Sequence reads were mapped to the *M*. *tuberculosis* H37Rv genome using Bowtie [43]. Reads mapping to more than one location within the genome were excluded from further analysis. BEDTools was used to determine counts for each coding sequence in the *M*. *tuberculosis* H37Rv genome [44]. The above analyses were carried out within the web-based Galaxy platform [45, 46, 47]. Differential expression analysis using the output count data was carried out in R with the Bioconductor software package, edgeR [48]. Genes demonstrating ≥2 fold differential expression between Δ*csoR* and wild type strains, and having an FDR < 0.05 were considered to be differentially expressed. Confirmation of 10 differentially expressed genes was carried out by qRT-PCR as detailed above.

### Whole genome sequencing

Genomic DNA was extracted as described previously [49]. Quality was confirmed by gel electrophoresis and measuring absorbance at 260nm and 280nm. Samples were submitted to the University of Wisconsin Biotechnology Center’s DNA Sequencing Facility where they were prepared for sequencing using the NEBNext® Ultra™ DNA Library Prep Kit for Illumina® (New England Biolabs, Ipswich, MA). The samples were run on an Illumina MiSeq™ to generating 300bp paired end reads. Base calling was done in CASAVA 1.8.2. Reads were mapped to the *M*. *tuberculosis* H37Rv genome (NC_000962.3) using the CLC Genomics Workbench 8.0.1 (CLC bio, Aarhus, Denmark). SNPs were detected using the Fixed Ploidy Variant Detection tool, and larger genomic variations were detected using the InDels and Structural Variants tool with the same software.

### Statistical analysis

For large scale differential expression analysis, false discovery rates were calculated using the Benjamini and Hochberg algorithm [50] with a cutoff for significance at FDR < 0.05. For all other experiments, statistical significance was determined using Student’s *t*-test with cutoff value of *P* < 0.05.

## Supporting Information

S1 Fig Growth kinetics of Δ*csoR* under copper stress. Growth of *M*. *tuberculosis* H37Rv (circles) and Δ*csoR* (squares) over the course of 15 days in Sauton’s media left with 50μM CuCl_2_ from **(A)** log stage or **(B)** stationary stage inocula. Shown are one of two similar biological replicates with error bars representing standard deviation.
(TIF)

S2 Fig Growth inhibition of wild type H37Rv, Δ*csoR*, and complement strains by CuCl_2_. *M*. *tuberculosis* H37Rv (red circles), Δ*csoR* (green squares), and complement (blue triangles) strains were exposed to two-fold dilutions of CuCl_2_ in Sauton’s broth from 16μM to 4000μM. Percent reduction of Alamar blue reagent as compared to untreated wells was used to measure growth. Data are representative of two biological replicates.
(TIF)

S3 Fig Growth of Δ*csoR* and wild type H37Rv in the spleen and liver during mouse infection. Groups of BALB/c mice were infected by aerosol route with either *M*. *tuberculosis* H37Rv (filled circles) or Δ*csoR* (open squares). Shown are CFU/g of **(A)** spleen or **(B)** liver for individual mice over the course of 25 weeks representative of one of two similar experiments. Dashed lines indicate the limit of detection (500 and 100 CFU/g respectively) for each experiment. Data and mean shown are composites from two independent experiments. **(C)** Total grams body weight were also recorded and represent a single experiment. *P = 0.005.
(TIF)

S4 Fig Histopathology scores of wild type and Δ*csoR* in the lung, spleen, and liver during mouse infection. Groups of BALB/c mice were infected by aerosol route with either *M*. *tuberculosis* H37Rv (filled circles) or Δ*csoR* (open squares). Shown are histopathology scores (0, absent; 1, minimal; 2, mild; 3, moderate; 4, severe; 5, massive) for **(A)** granulomatous inflammation and **(B)** bronchiole-associated lymphoid tissue in the lungs; (**C)** granulomatous inflammation and **(D)** lymphocytic inflammation in the liver; and **(E)** granulomatous inflammation and **(F)** follicular atrophy in the spleen. Data represent all readings for three animals per time point in each group.
(TIF)

S5 Fig RT-PCR confirmation of RNA-Seq results. qRT-PCR was used to confirm the differential expression of 10 genes, 6 induced and 4 repressed, identified from the RNA-Seq experiment. Shown is the fold change of gene expression in the Δ*csoR* strain compared to wild type for the RNA-Seq data (black) and the qRT-PCR confirmation (white). For qRT-PCR data, the means of two biological replicates are shown with error bars representing the standard deviations.
(TIF)

S6 Fig Isoniazid resistance in Δ*csoR*. **(A)** 6mm discs were impregnated with 160ng isoniazid and placed on 7H10 agar plates with ADC spread with 100ul late log stage culture of *M*. *tuberculosis* H37Rv (red) or Δ*csoR* (green). Once a lawn of growth was detected, the radius of the zone of inhibition around the disc was measured. Error bars indicate the standard deviation. Data are representative of two biological replicates. **(B)** *M*. *tuberculosis* H37Rv (red circles) and Δ*csoR* (green squares) were exposed to two-fold dilutions of isoniazid in 7H9 broth with ADC from 3ng/ml to 80ng/ml. Percent reduction of Alamar blue reagent as compared to untreated wells was used to measure growth. Data are representative of two biological replicates.
(TIF)

S7 Fig Assessing copper levels in the cell using *mymT* transcription levels as an indirect indicator. Expression levels of *mymT* as determined by qRT-PCR analysis of samples H37Rv, Δ*csoR*, or Δ*csoR*::*csoR* left untreated (0μM) (black) or stressed with 50μM (striped) or 500μM (white) CuCl_2_. Fold change is shown as expression levels of each gene relative to expression levels in untreated wild type culture after normalization to *sigA* expression levels. Data represent one of two similar biological replicates. Error bars represent the standard error of the mean from two technical replicates.
(TIF)

S1 Table Total RNA-Seq reads mapping to the M. tuberculosis H37Rv genome. (XLSX)

S2 Table All significantly differentially expressed genes in *M*. *tuberculosis* Δ*csoR* as compared to H37Rv wild type. (XLSX)
