# Supplementary figures and images for: CsoR Is Essential for Maintaining Copper Homeostasis in Mycobacterium tuberculosis
Source: PLoS One. 2016 Mar 21;11(3):e0151816. doi: 10.1371/journal.pone.0151816 (PMC4801387; doi:10.1371/journal.pone.0151816)

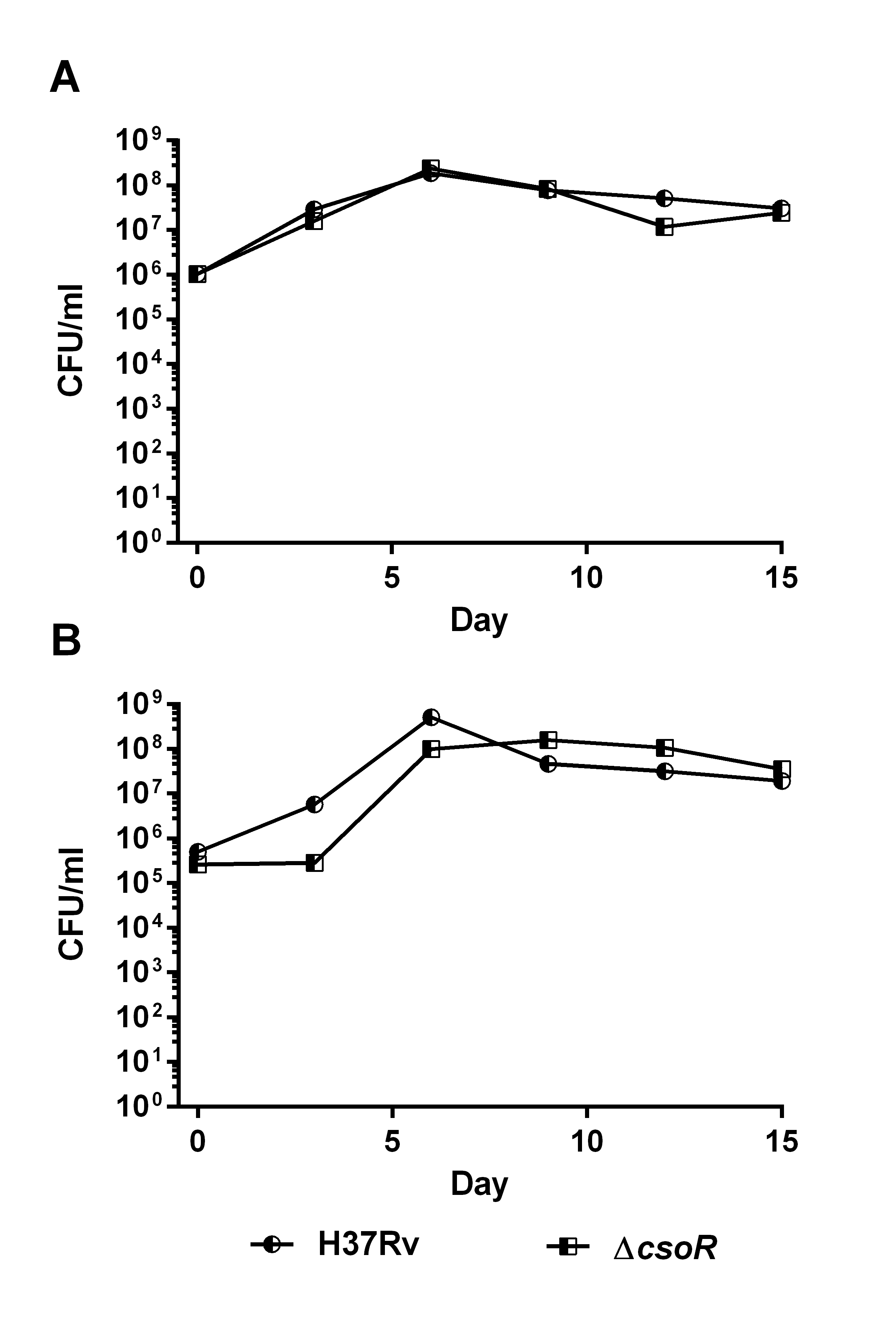

Supplement: S1 Fig — Growth of M. tuberculosis H37Rv (circles) and ΔcsoR (squares) over the course of 15 days in Sauton’s media left with 50μM CuCl2 from (A) log stage or (B) stationary stage inocula. Shown are one of two similar biological replicates with error bars representing standard deviation. (TIF) [file pone.0151816.s001.tif]

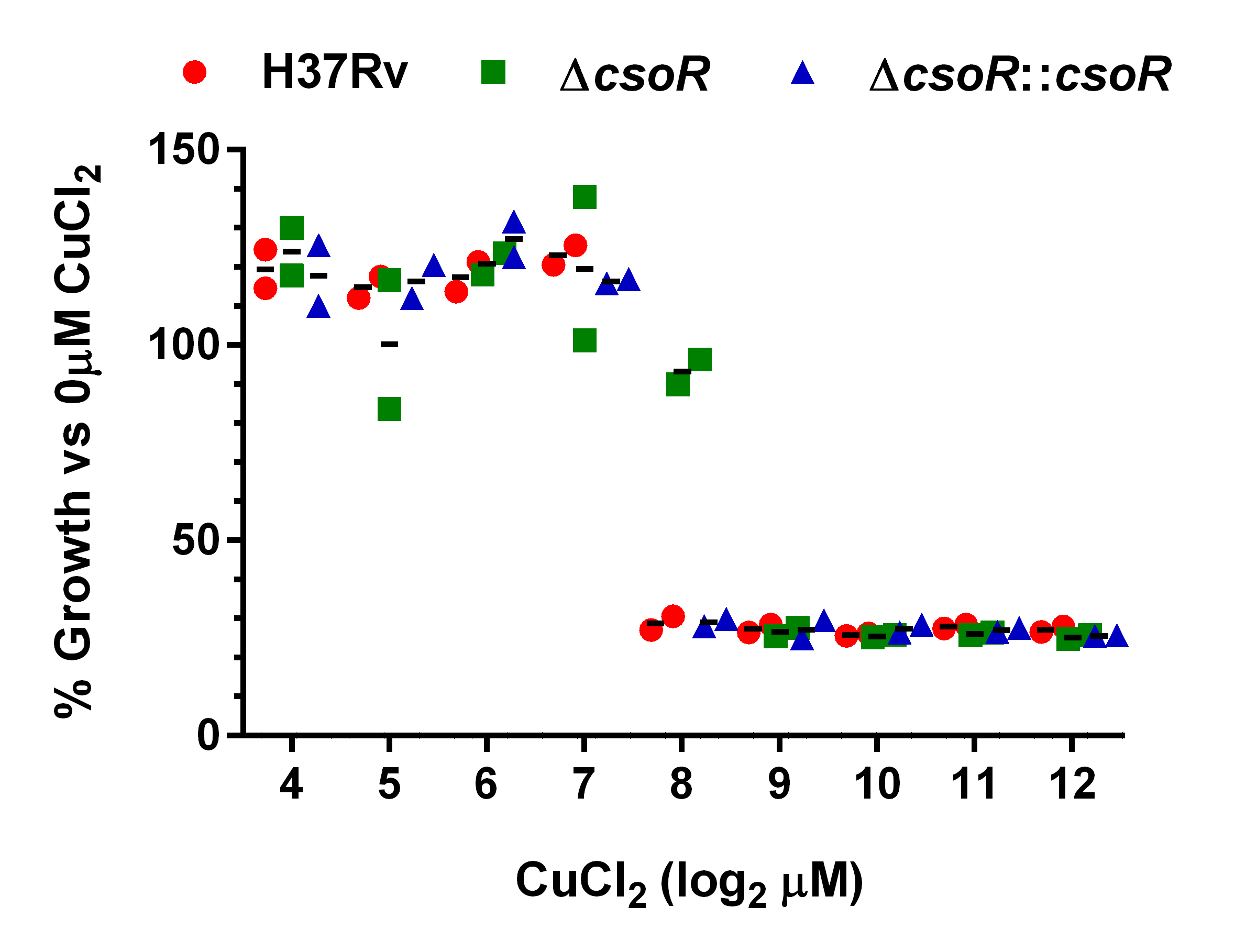

Supplement: S2 Fig — M. tuberculosis H37Rv (red circles), ΔcsoR (green squares), and complement (blue triangles) strains were exposed to two-fold dilutions of CuCl2 in Sauton’s broth from 16μM to 4000μM. Percent reduction of Alamar blue reagent as compared to untreated wells was used to measure growth. Data are representative of two biological replicates. (TIF) [file pone.0151816.s002.tif]

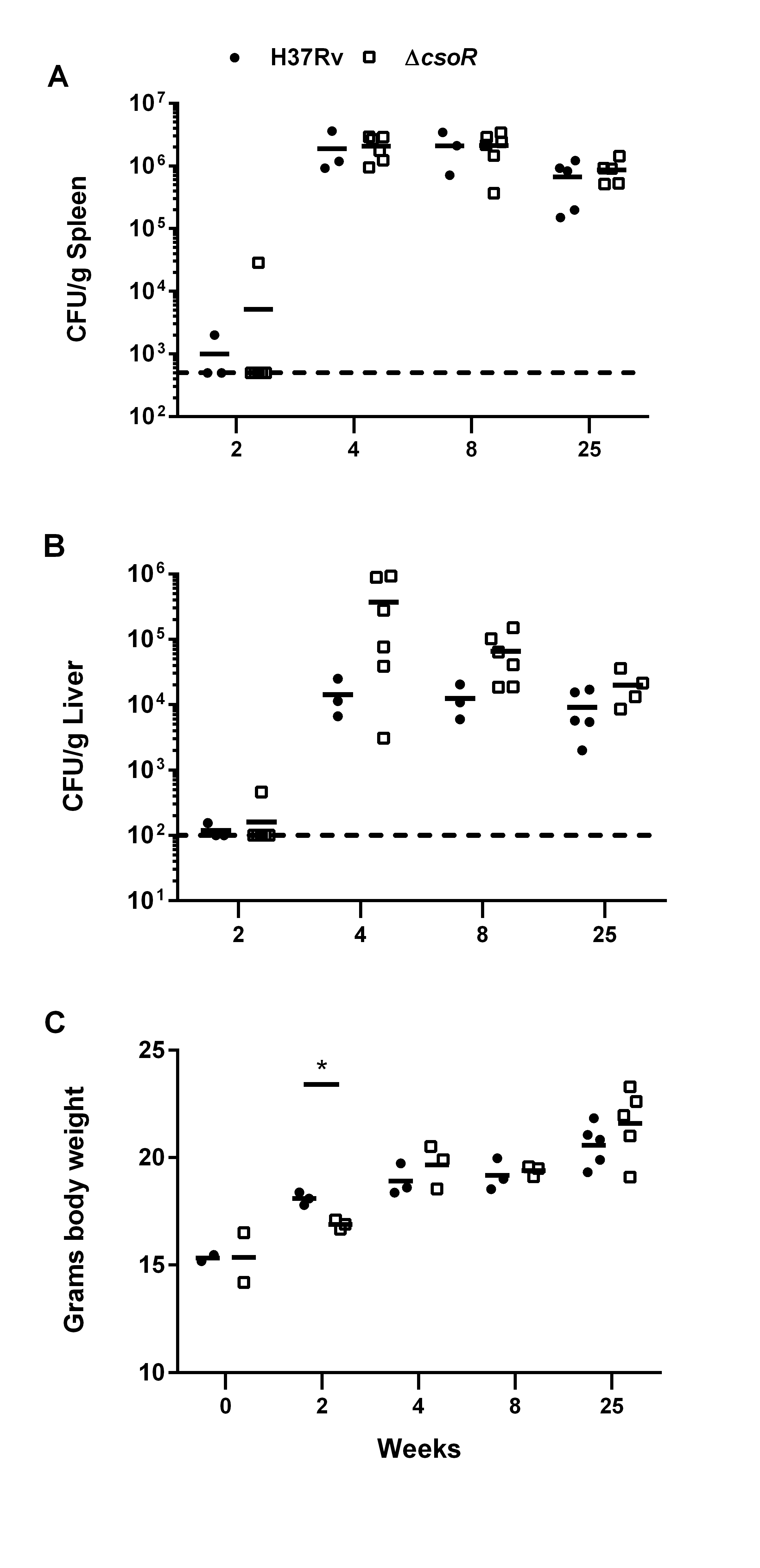

Supplement: S3 Fig — Groups of BALB/c mice were infected by aerosol route with either M. tuberculosis H37Rv (filled circles) or ΔcsoR (open squares). Shown are CFU/g of (A) spleen or (B) liver for individual mice over the course of 25 weeks representative of one of two similar experiments. Dashed lines indicate the limit of detection (500 and 100 CFU/g respectively) for each experiment. Data and mean shown are composites from two independent experiments. (C) Total grams body weight were also recorded and represent a single experiment. *P = 0.005. (TIF) [file pone.0151816.s003.tif]

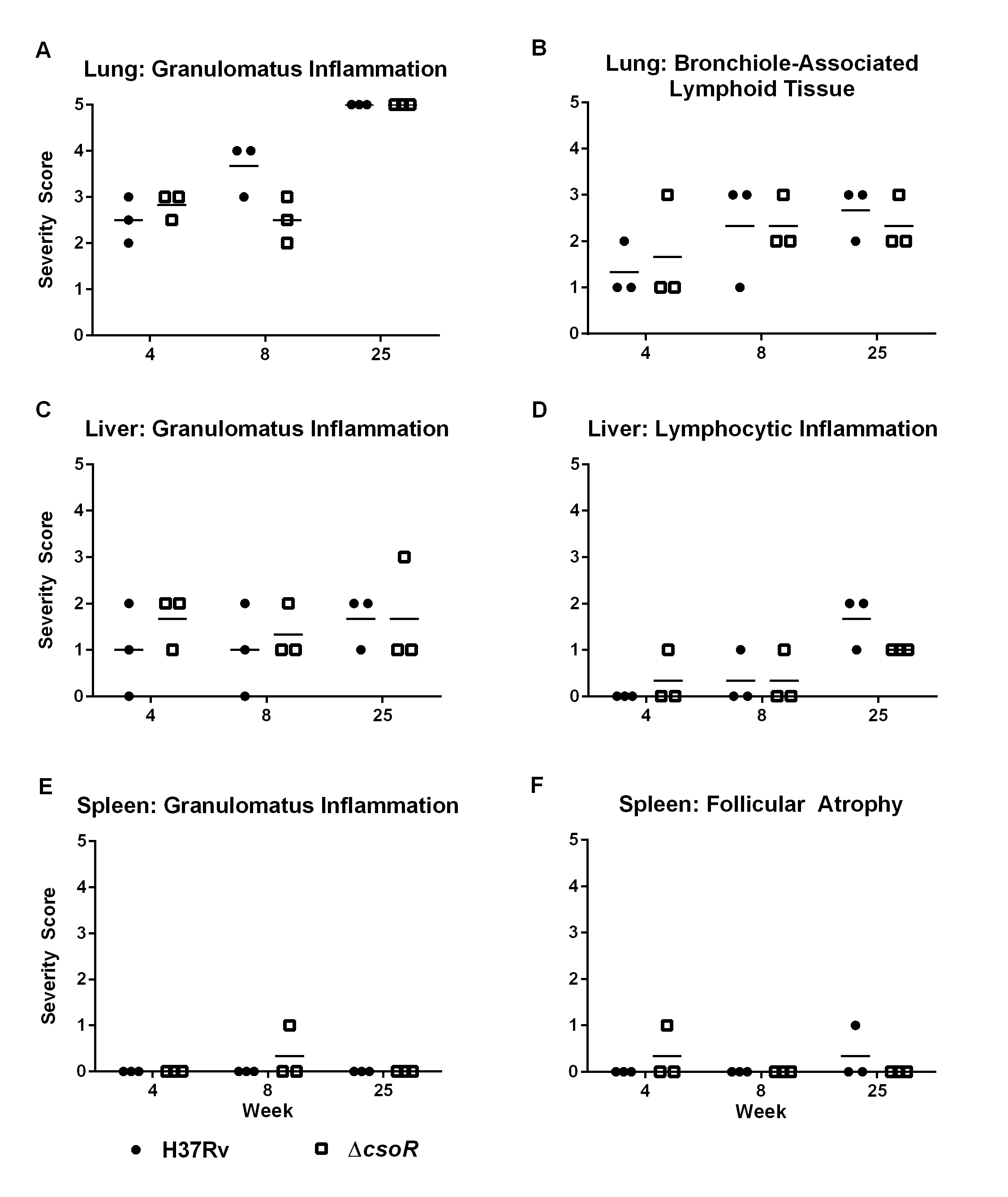

Supplement: S4 Fig — Groups of BALB/c mice were infected by aerosol route with either M. tuberculosis H37Rv (filled circles) or ΔcsoR (open squares). Shown are histopathology scores (0, absent; 1, minimal; 2, mild; 3, moderate; 4, severe; 5, massive) for (A) granulomatous inflammation and (B) bronchiole-associated lymphoid tissue in the lungs; (C) granulomatous inflammation and (D) lymphocytic inflammation in the liver; and (E) granulomatous inflammation and (F) follicular atrophy in the spleen. Data represent all readings for three animals per time point in each group. (TIF) [file pone.0151816.s004.tif]

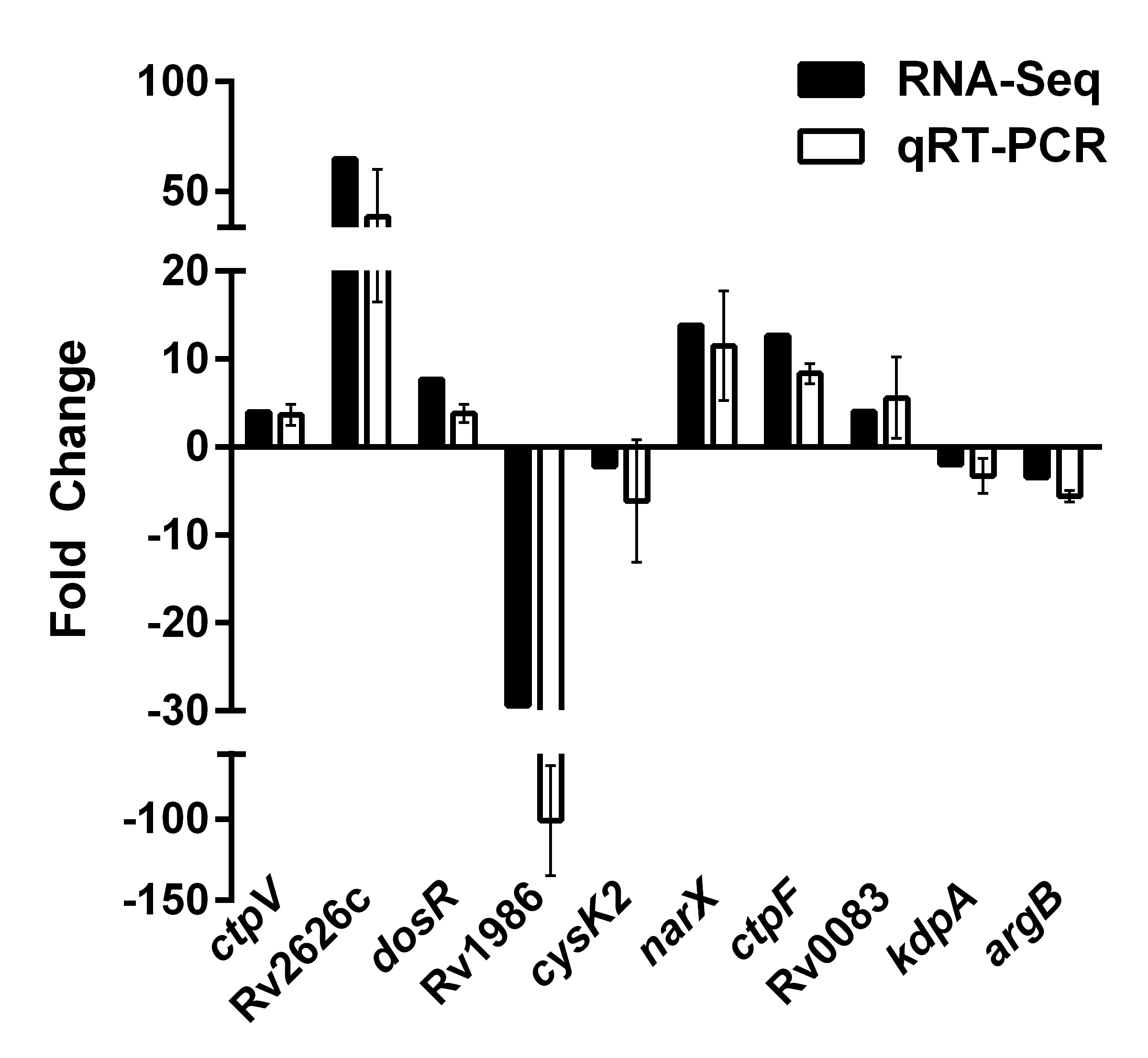

Supplement: S5 Fig — qRT-PCR was used to confirm the differential expression of 10 genes, 6 induced and 4 repressed, identified from the RNA-Seq experiment. Shown is the fold change of gene expression in the ΔcsoR strain compared to wild type for the RNA-Seq data (black) and the qRT-PCR confirmation (white). For qRT-PCR data, the means of two biological replicates are shown with error bars representing the standard deviations. (TIF) [file pone.0151816.s005.tif]

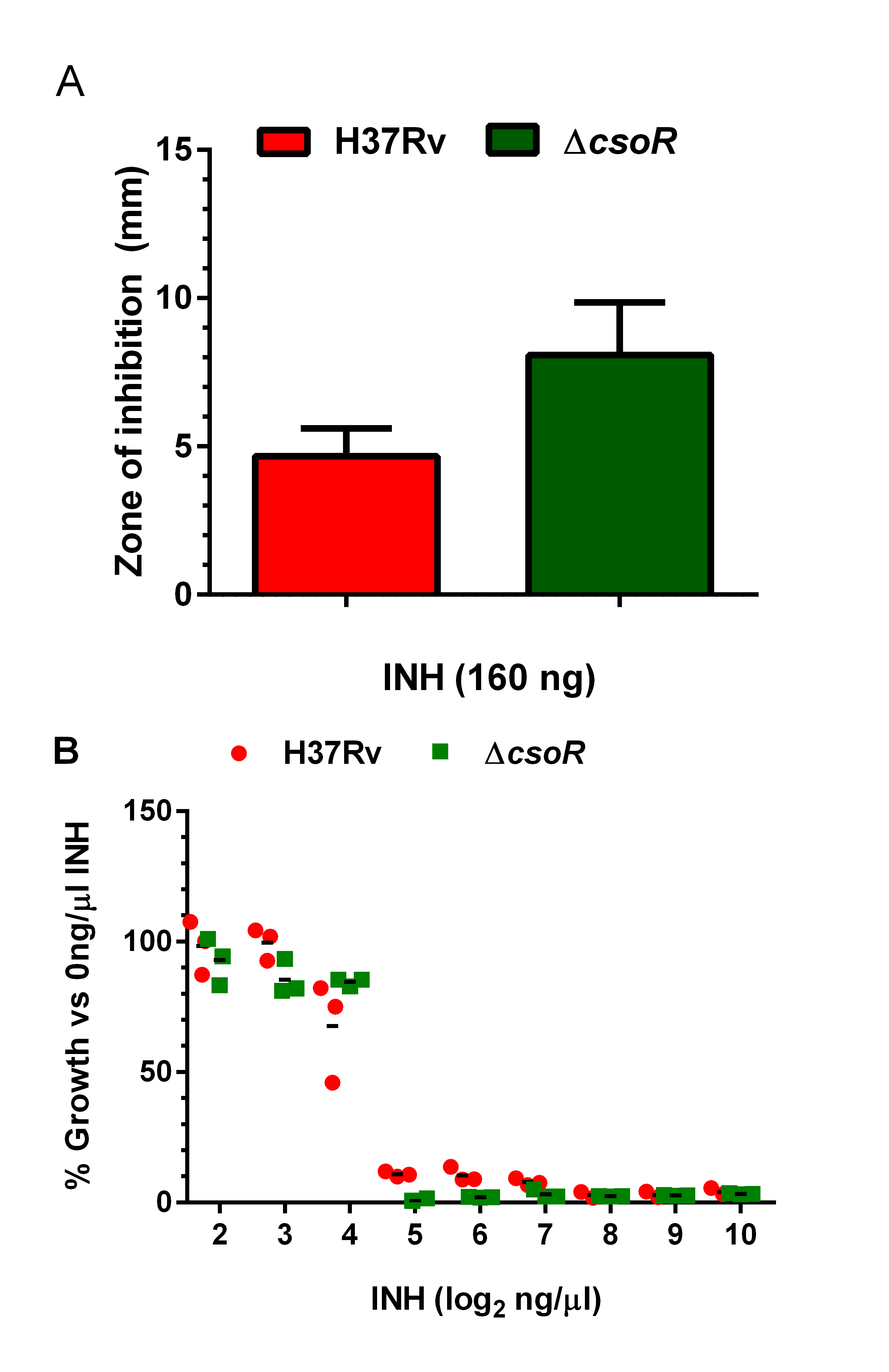

Supplement: S6 Fig — (A) 6mm discs were impregnated with 160ng isoniazid and placed on 7H10 agar plates with ADC spread with 100ul late log stage culture of M. tuberculosis H37Rv (red) or ΔcsoR (green). Once a lawn of growth was detected, the radius of the zone of inhibition around the disc was measured. Error bars indicate the standard deviation. Data are representative of two biological replicates. (B) M. tuberculosis H37Rv (red circles) and ΔcsoR (green squares) were exposed to two-fold dilutions of isoniazid in 7H9 broth with ADC from 3ng/ml to 80ng/ml. Percent reduction of Alamar blue reagent as compared to untreated wells was used to measure growth. Data are representative of two biological replicates. (TIF) [file pone.0151816.s006.tif]

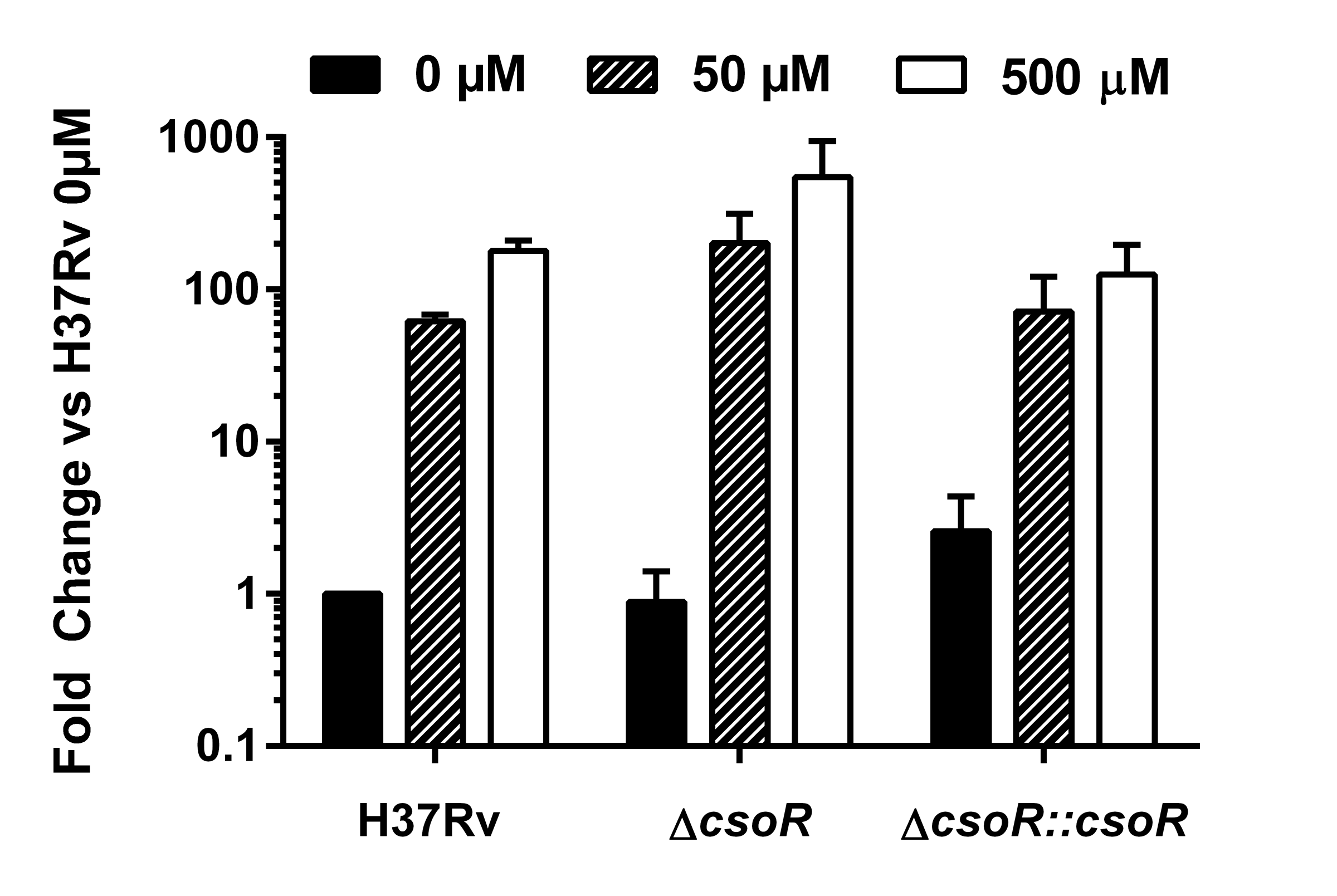

Supplement: S7 Fig — Expression levels of mymT as determined by qRT-PCR analysis of samples H37Rv, ΔcsoR, or ΔcsoR::csoR left untreated (0μM) (black) or stressed with 50μM (striped) or 500μM (white) CuCl2. Fold change is shown as expression levels of each gene relative to expression levels in untreated wild type culture after normalization to sigA expression levels. Data represent one of two similar biological replicates. Error bars represent the standard error of the mean from two technical replicates. (TIF) [file pone.0151816.s007.tif]
